# Swimming training attenuates the decrease of calcium responsiveness in female infarcted rats

**DOI:** 10.3389/fphys.2022.923603

**Published:** 2022-08-22

**Authors:** Leslie Andrews Portes, Alexandra Alberta dos Santos, Carlos Roberto Padovani, Natália Cristina de Oliveira, Andrey Jorge Serra, Paulo J. F. Tucci

**Affiliations:** ^1^ Professor at UNASP, Researcher at LAFEX, Laboratory of Exercise Physiology, Adventist University of Sao Paulo, Sao Paulo, Brazil; ^2^ Laboratory of Cardiac Physiology and Cardiovascular Physiopathology, Federal University of Sao Paulo, Sao Paulo, Brazil; ^3^ Department of Biostatistics, Bioscience Institute, UNESP-State University of Sao Paulo, Sao Paulo, Brazil; ^4^ Master Degree Program in Health Promotion at UNASP-Adventist University of São Paulo, Sao Paulo, Brazil

**Keywords:** myocardial infarction, rats, inoprotpism, lusitropism, calcium responsivity, swimming exercise, exercise training

## Abstract

**Aim:** To evaluate the influence of swimming training on calcium responsiveness of the myocardium of rats with different infarction sizes (MI).

**Method:** female Wistar rats, sedentary sham (SS = 14), sedentary moderate MI (SMI = 8) and sedentary large MI (SLI = 10) were compared to trained sham (TS = 16), trained moderate MI (TMI = 9) and trained large MI (TLI = 10). After 4 weeks of MI, the animals swam for 60 min/day, 5 days/week, for additional 8 weeks. Papillary muscles of the left ventricle were subjected to different concentrations of extracellular calcium. Inotropism was evaluated through the developed tension (DT), the maximum positive value of the first temporal derivation (+Td/td) and the time to peak tension (TPT). Lusitropism was evaluated by the maximum negative value of the first temporal derivation (−Td/td) and time to 50% relaxation (50%TR). Statistical significance was determined using multivariate analysis of variance and a Hotelling T2 test for the absolute power values of all four extracellular calcium concentrations (*p* < 0.05).

**Results:** MI depressed inotropism (from 17% to 51%) and lusitropism (from 22% to 54%) of the sedentary rats, but exercise attenuated the losses, especially regarding + dT/dt, TPT, −dT/dt and 50%TR. Exercise attenuated the decrease in myocardial responsiveness, proportionally to the size of the MI.

**Conclusion:** Myocardial calcium responsiveness is favorably affected in animals with moderate and large MI after swimming exercise.

## Introduction

Physical exercise is one of the most important nonpharmacological therapies after myocardial infarction (MI), as it attenuates cardiac and myocardial remodeling, preserving systolic and diastolic functions (Bowles et al., 1992; Orenstein et al., 1995; Wisloff et al., 2002; Serra et al., 2008; Serra et al., 2010; Andrews Portes et al., 2009). The postulated mechanisms for these benefits include the attenuation of fetal β-myosin isoform expression (Orenstein et al., 1995), reduction of reactive oxygen species (Frederico et al., 2009), reduction of pro-inflammatory cytokines (Serra et al., 2010), and improved calcium sensitivity and kinetics of cardiomyocytes from infarcted hearts (Wisloff et al., 2002). Little is known about the effects of physical exercise on myocardial responsiveness, especially for hearts with different MI sizes (Bowles et al., 1992).

Bowles et al. (1992) subjected hearts from treadmill-trained male rats to 25 min of ischemia followed by 30 min of reperfusion and noticed better recovery of the systolic function, greater cardiac work and less diastolic stiffness, associated with greater responsiveness to extracellular calcium, compared to non-trained male rats. On the other hand, Nutter et al. (1981) found that exercise was detrimental to the contractile function of papillary muscles of the heart of healthy male rats, and this impairment was associated with depression of calcium responsiveness.

Since myocardial responsiveness to calcium is an important physiological determinant of the contractile mechanism, and that MI impairs this response (Fellenius et al., 1985; Sandmann et al., 1999), the present study evaluated the influence of aerobic physical exercise on papillary muscles of female hearts rats with moderate and large MI. This information will broaden the understanding of the effects of aerobic exercise on the myocardium of infarcted hearts. Our hypothesis was that aerobic swimming exercise favorably affects myocardial responsiveness to calcium.

## Methods

### Animals, myocardial infarction induction and experimental groups

Female Wistar rats weighing 170–190 g were housed under a 12/12 h dark/light cycle, at a temperature of 22°C−23°C and humidity of 54%–55%. The rats had free access to water and to a pellet rodent diet. MI was induced according to the procedure as described by Andrews Portes et al. (2009), and were anesthetized (Ketamine, 90 mg/kg and Xylazine, 10 mg/kg; intraperitoneally), intubated and ventilated (model 683, Harvard Apparatus, 2.0 ml, 80 strokes/min). A left thoracotomy was performed, and a 6.0 silk thread was permanently tied around the left anterior descending coronary artery. In the sham rats, coronary occlusion was not undertaken. After the heart was quickly returned to the thorax, a purse string suture allowed chest closure and the rats remained sedentary for 4 weeks.

After 4 weeks, transthoracic Doppler echocardiography evaluation was performed under the same anesthesia using a 12-MHz transducer (Sonos-5500, Hewlett-Packard, Andover, Massachusetts) to determine infarct size, as described elsewhere (Cury et al., 2005). MI smaller than 20% were excluded. According to the MI size, the MI rats were grouped as: sedentary moderate infarct (SMI: *n* = 8), trained moderate infarct (TMI: *n* = 9), sedentary large infarct (SLI: *n* = 10) and trained large infarct (TLI: *n* = 8). A moderate infarction was considered for rats presenting a MI scar occupying 20%–39% of the LV and a large MI for rats presenting a MI scar equal to or larger than 40% of the LV. In addition, two sham groups were studied: sedentary sham (SS: *n* = 14), and trained sham (TS: *n* = 15). After the protocol and heart dissection, infarct size was confirmed by planimetry. The left ventricle (LV) was isolated and unrolled, and straight incisions allowed the domelike LV shape to lie flat when placed over a thin glass plate. Using transillumination, the contours of the infarcted area and of the entire left ventricle mass were traced on a transparent acetate plate and the areas measured using Sigma Scan Pro 5.0 (Systat Software Inc., Richmond, California, United States). The infarct sizes were expressed as a percentage of the LV area.

The rats were cared for in compliance with the “Principles of Laboratory Animal Care” formulated by the National Institutes of Health (National Institutes of Health publication no. 96-23, revised, 1996) and the protocol (#16/2003) was approved by the Institutional Ethics Committee of Escola Paulista de Medicina, Federal University of Sao Paulo, Sao Paulo, Brazil.

### Exercise training protocol

The exercise protocol was in conformity with the “American Physiological Society: Resource Book for the Design of Animals Exercise Protocols” (Kregel, 2006). Swimming training was initiated 4 weeks after coronary occlusion and was performed in a container (depth of 80 cm) filled with tap water kept at 32°C–34°C by a feed-back controlled electric heating coil. The water was maintained in continuous turbulence to provide a continuous exercise. For adaptation, training was limited to 10 min on the first day and increased by 10 min each day until day 6. Training was then continued for a total period of 8 weeks, 60 min/day and 5 days/week. Ten to 12 rats swam simultaneously. Rats were toweled dry after each swimming session before they were returned to their cages. Rats randomized to sedentary conditions did not swim during the 8 weeks. This exercise protocol corresponds to about 75% of the maximal VO_2_ (McArdle, 1967) and has already been shown to be favorable to myocardial inotropism and lusitropism (Andrews Portes et al., 2009).

### Myocardial studies by papillary muscles

After 13 weeks of the MI or sham procedures (4 weeks after surgery, 1 week for adaptation and 8 weeks for swimming training), under anesthesia, the hearts were quickly removed and placed in oxygenated Krebs-Henseleit solution at 30°C. A papillary muscle was carefully dissected from the LV, mounted between two spring clips, and placed vertically in a chamber containing Krebs-Henseleit solution at 28°C, oxygenated with 100% O_2_, and pH 7.40 ± 0.02. The composition of the Krebs-Henseleit solution was as follows (in mM): 132 NaCl, 4.69 KCl, 1.5 CaCl_2_, 1.16 MgSO_4_, 1.18 KH_2_PO_4_, 5.50 C_6_H_12_O_6_, 20 HEPES, pH 7.40. The lower spring clip was attached to the bottom of the chamber and the upper spring clip was connected by a thin steel wire to an isometric transducer (GRASS model FT03E) connected to a micrometer for adjustment of muscle length. The preparations were stimulated 12 times/min with 5 ms square-wave pulses through parallel platinum electrodes, at voltages which were ∼10% greater than the minimum stimulus required to produce a maximal mechanical response. After a 60 min period, during which the preparation was permitted to contract isotonically under light loading conditions (0.4 g), the papillary muscle was loaded to contract isometrically during 15 min and, thereafter, was stretched to the apices of their length-tension curves (L_max_). The mechanical behavior of papillary muscles were evaluated in baseline calcium condition (1.5 mmol/L) and in three different other extracellular calcium concentration [(Ca^2+^)_o_, in mmol/L]: 0.5, 1.0 and 2.0. The 1.5 mmol/L was reassumed for each (Ca^2+^)_o_. The contractile myocardial parameters were determined for each (Ca^2+^)_o_ after a stable period of 10 min. The following parameters were measured during the isometric contractions: peak developed tension (DT, g/mm^2^), resting tension (RT, g/mm^2^), maximum rate of developed tension (+dT/dt) and maximum rate of decline tension (–dT/dt), time to peak tension (TPT), and time from peak tension to 50% relaxation (TR50%). At the end of each experiment, muscle length at L_max_ was measured and the muscle between the two clips was blotted dry and weighed. The cross-sectional area was calculated from the muscle weight and length by assuming cylindrical uniformity and a specific gravity of 1.04. All experiments were carried out at 28°C (Andrews Portes et al., 2009).

### Statistical analysis

Statistical significance between groups was determined using multivariate analysis of variance (MANOVA) and a Hotelling T^2^ test for the absolute power values of all four extracellular calcium concentrations [(Ca^2+^)_o_]. Calcium responsiveness was determined from a linear regression analysis between the increase in the papillary muscle contractile parameters and the (Ca^2+^)_o_. The slope angles of the linear regression curves of each animal (slope) were analyzed by Kruskal-Wallis test, followed by Dunn’s posttest. Differences between the groups of animals in relation to the values of the slopes of the curves were considered statistically significant if *p* < 0.05. Statistical analysis was performed using SigmaStat version 3.5 for Windows.

## Results

### Myocardial infarction size

The myocardial infarctions sizes were: SMI: 31 ± 6%, TMI: 31 ± 4%, SLI: 47 ± 6% and TLI: 49 ± 11%.

### Papillary muscle data

The cross-sectional area (CSA) was higher (*p* = 0.016) in SMI (1.3 ± 0.3 mm^2^) and SLI (1.5 ± 0.5 mm^2^) in comparisons to the sham rats (SS: 0.9 ± 0.3 mm^2^) but was not significantly different (*p* = 0.083) in relationship to trained rats (TS: 0.9 ± 0.2 mm^2^ = TMI: 1.0 ± 0.4 mm^2^ = TLI: 1.2 ± 0.3 mm^2^).

**FIGURE 1:**
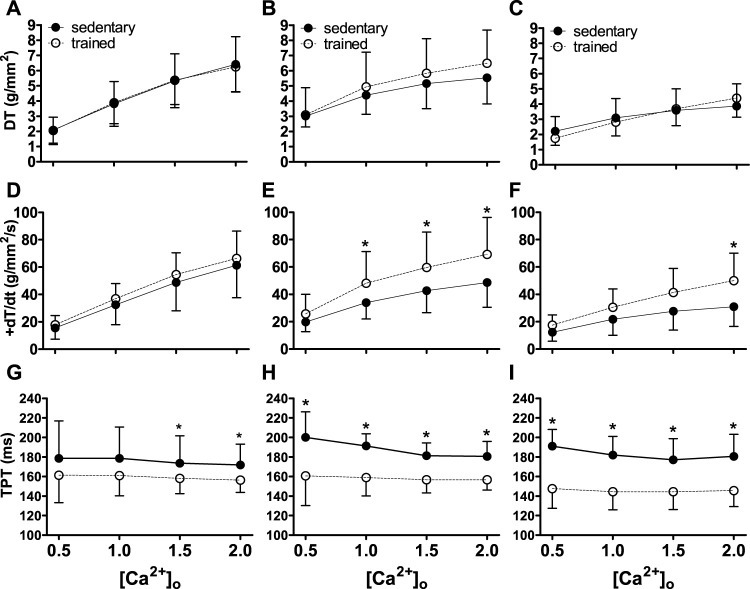
Inotropic function (DT, +dT/dt and TPT) of sedentary rats (solid symbols) and trained rats (empty symbols) with different myocardial infarction size after swimming exercise training. Inotropy was evaluated by different extracellular calcium concentrations [(Ca^2+^)_o_]. The myocardial infarction size: Sham operated **(A,D,G)**, Moderate MI **(B,E,H)**, and Large MI **(C,F,I)**. Comparisons between sedentary and trained rats: *p < 0.05. Values were expressed by mean ± SD.

The data (means ± SD) on contractile function are shown in Figure 1.

There was an increase (*p* < 0.001) in DT and +dT/dt as a function of the increase in (Ca^2+^)_o_, both in sedentary and trained animals (Figures 1A–F). Physical exercise attenuated MI losses in relation to + dT/dt (*p* < 0.05) in TMI and TLI animals. TPT was abbreviated in all exercised groups (*p* < 0.001) in relation to the respective sedentary ones (Figures 1G–I). These results indicate benefits of physical exercise in relation to myocardial inotropism, despite the size of the infarction.

**FIGURE 2:**
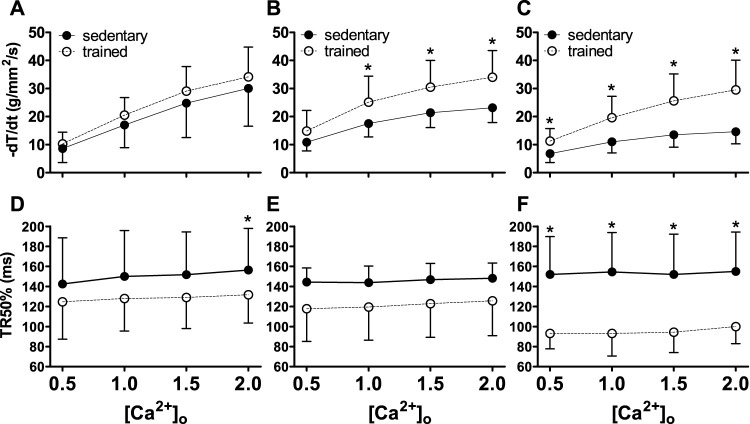
Lusitropic function (−dT/dt and TR50%) of sedentary rats (solid symbols) and trained rats (empty symbols) with different myocardial infarction size after swimming exercise training. Lusitropy was evaluated by different extracellular calcium concentrations [(Ca^2+^)_o_]. The myocardial infarction size: Sham operated **(A,D)**, Moderate MI **(B,E)**, and Large MI **(C,F)**. Comparisons between sedentary and trained rats: *p < 0.05. Values were expressed by mean ± SD.

Lusitropic function (−dT/dt and TR50%) were depicted in Figure 2, indicating an increase in the −dT/dt (Figures 2A–C) in relationship to (Ca^2+^)_o_ (*p* < 0.001). Swimming training attenuated MI effects in TMI and TLI. The TR50% (Figures 2D–F) was abbreviated in all trained rats (*p* < 0.001), notably in the TLI.

**FIGURE 3:**
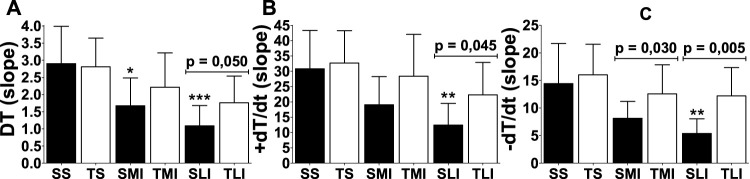
Responsiveness (slope) of inotropic (DT and +dT/dt) and lusitropic (−dT/dt) functions of papillary muscles of sedentary rats (closed bars) and trained rats (open bars) with different myocardial infarction size (sedentary sham: SS, trained sham: TS, sedentary moderate infarction: SMI, trained moderate infarction: TMI, sedentary large infarction: SLI, and trained large infarction: TLI) after swimming exercise training. Comparisons to respective sham animals: *p < 0,05, **p < 0,01 and ***p < 0.001.

The responsiveness was evaluated by the slope of curves (Figure 3) in DT, +dT/dt and −dT/dt. The greater the slope, the greater the myocardial responsiveness. Impairments in myocardial responsiveness triggered by MI were clearly attenuated by physical exercise (*p* < 0.05).

## Discussion

The present study adds strength to the evidence on the benefits of aerobic exercise for the hearts of rats with heart failure due to MI, providing evidence that improvements in systolic and diastolic function are related to the maintenance of myocardial responsiveness to increased (Ca^2+^)_o_ in cardiomyocytes, even in hearts with large MI (≥45% of the left ventricle).

Physical exercise by swimming is added to other forms of aerobic exercise or physical activity, such as treadmill exercise (Molé, 1978; Schaible and Scheuer, 1979; Wisloff et al., 2002), with some advantages. For instance, in some studies, myocardial hypertrophy aroused by treadmill exercise would be related to a reduction in body weight and not to an increase in cardiac mass (Molé, 1978), or to a true increase in cardiac mass (Schaible and Scheuer, 1979), though others (Wisloff et al., 2002) verified true myocardial hypertrophy, with an increase in length and width of cardiomyocytes. With or without myocardial hypertrophy, treadmill exercise resulted in improvement in cardiac or myocardial function.

There are concerns about the possibility that during the swimming exercise the animals experience episodes of hypoxia, or that they aspirate water, or even that they suffer cases of drowning, which would increase the risk of pulmonary congestion, aggravating the effects of heart failure resulting from MI. Some studies, in fact, observed an increase in pulmonary water content in animals with heart failure due to MI and exercised on a treadmill (Jain et al., 2000; Helwig et al., 2003), indicating that physical exercise worsened the effects of heart failure due to MI. However, data from our group (Portes and Tucci, 2006; Andrews Portes et al., 2009) and Veiga et al., 2019 revealed that swimming exercise did not worsen pulmonary congestion in rats with large MI (Veiga et al., 2019), or even attenuated the pulmonary water content (Portes and Tucci, 2006; Andrews Portes et al., 2009).

The use of female rats was due to previous data from our group indicating that morphological changes related to MI size and diastolic and systolic diameters, as well as functional data related to systolic (change in fractional area), and diastolic (E and A waves, and E/A ratio) functions, aroused by acute myocardial infarction, do not differ according to the animal’s gender (Antonio et al., 2015), with the advantage of virtually excluding the effects of testosterone on muscle mass and performance in the exercises.

Another important aspect to be previously considered refers to the different MI sizes. It has been widely documented (Pfeffer et al., 1979; Fletcher et al., 1981; Pfeffer et al., 1991) that rats with small MI (≥5% to <30%), moderate MI (≥30% to <45%) and large MI (≥45%) exhibit progressive and proportional impairment of systolic and diastolic function. While animals with small MI do not exhibit discernible hemodynamic impairments related to cardiac pumping capacity and pressure generation, animals with moderate MI exhibit reduced flow and pressure generation indices, and animals with large MI exhibit congestive heart failure associated with high diastolic filling pressures, reduced cardiac output, and minimal ability to respond to increased preload and afterload (Pfeffer et al., 1979). Data from our group agree with previous studies that animals with small MI (4% to <30%), medium MI (≥30% to <40%) and large MI (≥40%) also exhibited left ventricular dilatation, reduced systolic and diastolic function (Nozawa et al., 2006). Our group also observed that with the increase in the size of the MI, there was a worsening of pulmonary congestion, hypertrophy of the right and left ventricles, damage to the positive and negative derivatives, and the times for peak tension and relaxation of the papillary muscles (Andrews Portes et al., 2009). For all these reasons, the expectation in the present study was that myocardial responsiveness to calcium would be depressed proportionally to the size of the MI and that physical training by swimming would attenuate these losses. However, previous information on physical exercise and myocardial responsiveness are contradictory.

Nutter et al. (1981), for example, studied healthy animals and found impairments in the contractile function of papillary muscles of hearts submitted to physical training on a treadmill, both in terms of calcium responsiveness, norepinephrine responsiveness and the Frank-Starling mechanism. Authors were unable explain these findings, as there was a lack of evidence of pathological structural changes, edema, and left ventricular connective tissue hyperplasia.

Bowles et al. (1992) evaluated calcium responsiveness of treadmill-exercised rat hearts in healthy animals using the Langendorff model, before, after 25 min of ischemia and after 30 min of reperfusion. Sedentary and trained animals did not differ in cardiac and hemodynamic function in the pre-ischemia phase, indicating no influence of physical training, except that the maximum rate of ventricular pressure change (+dP/dt) of the trained group was worse than that of the sedentary ones. Still on the pre-ischemia phase, as a function of increased calcium concentrations, hearts from trained rats exhibited worse cardiac output and maximal systolic pressure than sedentary rats at lower concentrations (0.50–0.75 mM), and no difference at concentrations of 1.5 mM at 3.0 mM. In the post-ischemia phase, coronary flow, aortic flow and cardiac output of trained rats were better than those of sedentary rats, but not aortic pressure, systolic pressure, diastolic pressure, +dP/dt and −dP/dt. Still regarding the post-ischemia phase, only systolic pressure was significantly higher in the trained rats as a function of the increase in (Ca^2+^)_o_, but there were no differences in calcium sensitivity between sedentary and trained rats. Bowles et al. (1992) attributed the slight effects of training on systolic pressure responsiveness to calcium to the preservation of phosphocreatine levels, ATP and total nucleotides, and lower rate of AMP.

Fellenius et al. (1985) are among the first to show that MI impairs cardiac responsiveness to calcium. After coronary occlusion, infarct sizes ranged from 20% to 25% of the left ventricle and were therefore considered small. Both control and MI hearts exhibited increase in systolic pressure as a function of the increase in (Ca^2+^)_o_, but the MI exhibited peak systolic pressure at each (Ca^2+^)_o_ reduced by almost 50% when compared to controls. These authors did not provide a direct explanation for the decrease in cardiac responsiveness to the increase in (Ca^2+^)_o_, but they attribute these losses to the decrease in sarcolemma calcium channels and/or the reduction in calcium affinity resulting from MI.

Wisloff et al. (2002) evaluated isolated cardiomyocytes from infarcted female rats exercised on a treadmill and observed that, with increasing stimulation frequency, the maximum shortening of the trained cells occurred with intracellular calcium rates approximately 50% lower than their respective sedentary controls. Authors interpreted this phenomenon as an increase in calcium sensitivity (between 5% and 35% greater sensitivity), induced by physical exercise. They also noticed a slight lusitropic effect of physical exercise, with a reduction of 50% in the time to drop intracellular calcium. This increased sensitivity of cardiomyocytes to calcium was associated with increased expression of the Na^+^/Ca^2+^ exchanger (NCX), SERCA2a, and phospholamban phosphorylation. Such changes would explain the faster removal of cytosolic calcium, improving relaxation and contraction (Zhang et al., 2000; Medeiros et al., 2004; Rolim et al., 2007). Additionally, the improvement in metabolic profile with training could explain the increase in the rate of troponin I phosphorylation, resulting in a higher rate of uncoupling between myosin and actin, increasing the rate of relaxation (Belin et al., 2006).

The present study confirms that MI impairs myocardial responsiveness to calcium (Fellenius et al., 1985; Wisloff et al., 2002), but clearly demonstrates that physical exercise attenuates these impairments (Wisloff et al., 2002). Additionally, it indicates that the myocardium of hearts with infarctions greater than 45% of the left ventricle benefits from aerobic exercise, as indicated by the greater responsiveness to calcium in inotropic and lusitropic maneuvers. These benefits would explain, in part, the positive effects of physical exercise on hearts with MI.

The main strengths of the present study are related to the use of multicellular preparations by means of papillary muscles, which allows studying the intimacy of myocardial mechanics and its responses to variations in (Ca^2+^)_o_. Another aspect is related to the use of physical exercise by swimming. This form of aerobic exercise mobilizes large muscle mass and corresponds to an intensity of approximately 75% of maximum oxygen consumption (McArdle, 1967) and eliminates the stress due to the use of electrical stimulation so that the animal remains active on the treadmill.

The present study was limited to assessing mechanical function. In the future, it would be desirable to associate myocardial alterations with the various proteins related to the intracellular calcium and calcium transient through the sarcolemma.

The present study was also limited in not evaluating the physical capacity of the animals, since, based on previous studies (McArdle, 1967; Schaible and Scheuer, 1979; Musch et al., 1988), swimming without weights added to the body represent moderate to vigorous intensity, requiring between 60% and 75% of VO2 max. It is also known that swimming does not allow fine adjustments in exercise intensity as with the treadmill, unless weights are added to the animal’s body. Nevertheless, this form of exercise is very suitable for the purpose of providing aerobic stimulation to animals with cardiovascular diseases, such as congestive heart failure resulting from MI.

## Conclusion

MI impairs myocardial responsiveness to calcium proportionally to MI size. Aerobic exercise attenuates these damages, largely preserving the inotropic and lusitropic response of the myocardium. Even in hearts with MI close to 50%, the benefits of physical exercise were identified, indicating a potential mechanism by which hearts with large MI still meet organic demands and contribute to increased survival in these animals.

## Data Availability

The raw data supporting the conclusions of this article will be made available by the authors, without undue reservation.
